# Anisotropic run-and-tumble-turn dynamics†

**DOI:** 10.1039/d3sm00589e

**Published:** 2023-12-14

**Authors:** Benjamin Loewe, Timofey Kozhukhov, Tyler N. Shendruk

**Affiliations:** a SUPA, School of Physics and Astronomy, University of Edinburgh Peter Guthrie Tait Road Edinburgh EH9 3FD UK bloewe@ed.ac.uk

## Abstract

Run-and-tumble processes successfully model several living systems. While studies have typically focused on particles with isotropic tumbles, recent examples exhibit “tumble-turns”, in which particles undergo 90° tumbles and so possess explicitly anisotropic dynamics. We study the consequences of such tumble-turn anisotropicity at both short and long-time scales. We model run-and-tumble-turn particles as self-propelled particles subjected to an angular potential that favors directions of movement parallel to Cartesian axes. Using agent-based simulations, we study the effects of the interplay between rotational diffusion and an aligning potential on the particles' trajectories, which leads to the right-angled turns. We demonstrate that the long-time effect is to alter the tumble-turn time, which governs the long-time dynamics. In particular, when normalized by this timescale, trajectories become independent of the underlying details of the potential. As such, we develop a simplified continuum theory, which quantitatively agrees with agent-based simulations. We find that the purely diffusive hydrodynamic limit still exhibits anisotropic features at intermediate times and conclude that the transition to diffusive dynamics precedes the transition to isotropic dynamics. By considering short-range repulsive and alignment particle–particle interactions, we show how the anisotropic features of a single particle are inherited by the global order of the system. We hope this work will shed light on how active systems can extend local anisotropic properties to macroscopic scales, which might be important in biological processes occurring in anisotropic environments.

## Abstract 2

By modeling run-and-tumble-turn particles that can only travel and turn along the four Cartesian axes, we show how microscopic anisotropic features extend to macroscopic scales, and are inherited, in the interacting case, by global order.

## Graphical abstract

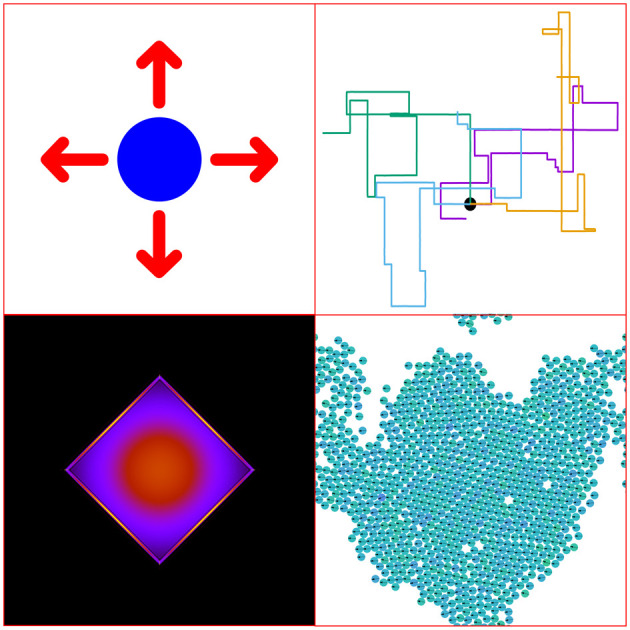

## 1 Introduction

Since its inception, the field of active matter has relied on idealized models of self-propulsion to study the transport properties of living systems.^1–3^ As persistent motion is often the main characteristic separating active particles from their passive counterparts, the unifying characteristic of these models is that particles are assumed to exert a propulsive force of constant magnitude but with a directionality that evolves in time through random processes.^3,4^ As such, the specifics of their stochastic directional processes set these models apart from one another.

On the one hand, there are models with rotational diffusion. In these models, the orientation of the particle's self-propulsion follows a Wiener process and can be written as a Langevin equation. Examples include Active Brownian Particles (ABP)^5–7^ and Active Ornstein–Uhlenbeck Particles,^8–11^ which have inspired analogies with quantum systems^12,13^ and successfully captured dynamic processes in flocks,^14–17^ suspensions of motile Janus particles,^18–20^ Quincke rollers,^21–23^ and cells within epithelial tissues.^24–28^ On the other hand, there exist run-and-tumble models.^29,30^ Run-and-tumble models mimic certain flagellar bacteria's characteristic motion (*E. coli*),^31–33^ particles randomly alternate between periods of constant self-propulsion (runs) and sudden sharp turning events (tumbles). The self-propulsion has a finite probability rate of instantaneously changing its direction, remaining constant otherwise. This time evolution makes writing a stochastic equation much harder than rotational diffusion. Recent attempts complement the Langevin equations with potentials that penalize deflections, thus retrieving effective run-and-tumble motion from a modified rotational diffusion.^34^ In addition, run-and-tumble motion has also been studied probabilistically using master equations,^35,36^ which can consider several variants of run-and-tumble motion that put different weights on possible turns relative to the current orientation and lead to analytical results for the moments and kurtosis of the distribution as functions of time. Although the distinction between ABP and run-and-tumble is inconsequential at long time scales,^37^ the different microscopic behaviors can lead to striking emergent phenomena, such as segregation of different active populations^38^ when interactions between particles are significant.

Both APB and run-and-tumble processes rely on isotropy and only penalize angular deviations from a particle's current orientation.^35,36^ Nevertheless, active particles rarely lie in isolation, often interacting with external fields that explicitly break rotational symmetry and orient the particle within space. Examples include magnetic fields in abiotic^39,40^ and biological elements,^41–43^ re-orientations caused by shear flow,^44^ chemotaxis in cells,^45^ bacteria^46,47^ and synthetic systems,^48^ swimmers in nematic liquid crystals^49^ and cells crawling through patterned substrates.^50–52^ Thus, such situations are expected to be anisotropic, depending on the particle's absolute orientation.

**Fig. 1:**
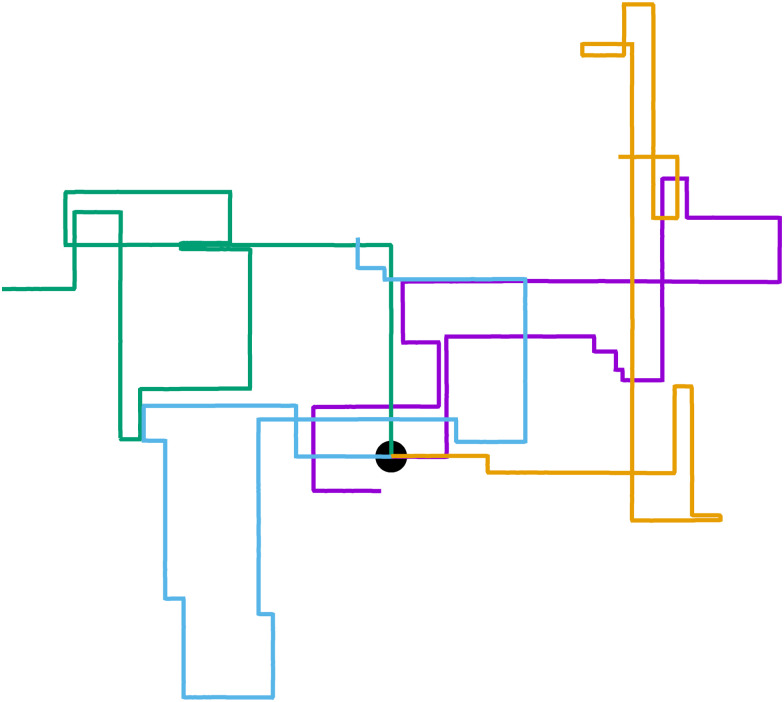
Run-and-tumble-turn particle trajectories (*v*_0_ = 1, *Γ* = 1, *D*_r_ = 7, *δ* = 1.3). The particles, which all have as a common starting point (the black circle), follow a run-and-tumble-turn process with sharp 90° turns.

We recently introduced one such example: a Janus colloid with mixed anchoring conditions (that is, prescriptions on the orientation that the nematic has at the surface of the colloid) embedded in an active nematic.^53^ This particle has planar anchoring (*i.e.*, the nematic lies parallel to the colloid's surface) on one side and homeotropic (*i.e.*, the nematic orientation is perpendicular to the colloid's surface) on the other. When immersed in an active nematic, the colloid effectively behaves as a self-propelled particle whose self-propulsion points either parallel or perpendicular to the local nematic director. Thus, this system represents an explicit realization of an effectively motile particle with externally imposed rotational symmetry breaking. The potential governing the colloid's orientation depends exclusively on the orientation itself and is entirely anisotropic. Activity-induced noise allows the colloid's orientation to hop from one potential well to another, leading to right-angled turns (Fig. 1). The colloid resembles an effective anisotropic run-and-tumble-turn particle, for which tumbles must be sudden 90° left or right turns. While this occurs in two dimensions, recent work has shown that active nematic droplets in three dimensions also exhibit pronounced right-angled turns.^54^

Inspired by the above example, we study the transport consequences of such a process, both analytically and computationally. To do so, we write a general Langevin equation for the particle's self-propulsion, which we use to simulate the run-and-tumble-turn dynamics (Section 2). We focus on 90° left or right tumble-turns as the most anisotropic tumble-turn case. Since we are motived by active biological systems, above 1 μm for bacteria and above 10 μm for epithelial cells, whose dynamics are dominated primarily by propulsive forces, we assume the model to be athermal, *i.e.*, neglect translational noise, but subject to orientational noise. To obtain analytical results, we approximate this to a Poisson process and write equations for the particle's probability density (Section 3). As the resulting model exhibits a hydrodynamical mode, we further coarse grain the model to obtain a modified, explicitly anisotropic diffusion equation (Section 4). By comparing these three descriptions, we find that, while highly anisotropic at short-time scales with solutions having a markedly square geometry, all three descriptions become effectively isotropic at long-time scales. While the Langevin and probabilistic description transition from self-propulsive at short-time scales to diffusive at long-time scales, the hydrodynamic theory is always diffusive, exhibiting that, although explicitly anisotropic, short wavelengths have a crucial role in shaping the propulsive behavior of the system.

Moreover, we demonstrate the dynamics are characterized by a strong separation of scales: while the transition to diffusive behavior is marked by the time scale determined by the tumble-turn rate, at short-time scales, the dynamics are governed by the relaxation time of the angular potential, resulting in a renormalization of the self-propulsion speed and a more or less noisy short-time trajectory. When observed in the time scales and length scales associated with the particles turning, all trajectories are indistinguishable, regardless of the details of the angular potential. This strongly supports the use of a probabilistic continuum model. We perform a spectral decomposition of the probabilistic description and study the dynamic interplay of different wavelengths in shaping the propulsive-to-diffusive transition. Complementing this with a study of the hydrodynamic limit reveals that this transition precedes the transition to isotropic dynamics, with anisotropicity persisting in the diffusive regime.

Finally, by including short-range repulsive and alignment interactions, we study the case of interacting run-and-turn-tumble particles (Section 5). We find that for sufficiently strong alignment interactions and high packing fractions, the four-fold symmetry of microscopic trajectories translates into the global orientational order of the system. Furthermore, this transition leads to a freezing of the angular sector of the energy landscape and states that are largely stationary in the center-of-mass frame. These results expand the literature on anisotropic self-propelled processes, exhibiting anisotropicity of subtle origins that differ from anisotropic diffusion. Moreover, they shed light on how active systems with inherent anisotropic local dynamics can extrapolate this property over macroscopic time and length scales, and by combining these features with particle–particle interactions, how they translate into a global order.

## 2 Colloid dynamics: Langevin equation

To model run-and-tumble-turn dynamics, we consider self-propelled particles traveling at a constant speed *v*_0_ with a direction specified by an orientation angle *ϕ* following a stochastic process. In contrast with the standard ABP, this process features an inherent anisotropy, incorporating a four-welled potential *V*(*ϕ*,*δ*), favoring traveling along the orthogonal axes, *x* and *y*. The dimensionless parameter *δ*, which runs from 0 to π/2, controls the width of the potential wells and, as such, quantifies how anisotropic this potential is. The equations governing these particles' position ***r***(*t*) and orientation *ϕ*(*t*) are given by

1 d***r*** = *v*_0_(cos(*ϕ*)***ê***_*x*_+ sin(*ϕ*)***ê***_*y*_)d*t*,

2
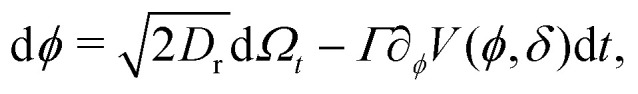
in which *Γ* is a rotational mobility, *D*_r_ a rotational diffusion coefficient and *Ω* denotes a Wiener process. Values detailed in Appendix A.

The angular potential *V*(*ϕ*,*δ*) is explicitly dimensionless, as we set its height as our unit of energy, *Γ* has units of inverse time. Inspired by the anisotropic steering dynamics exhibited by the active nematic Janus colloids,^53^ we consider the following rotational potential3
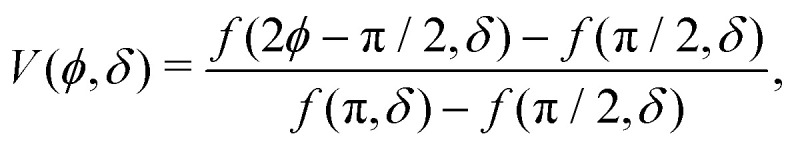
4
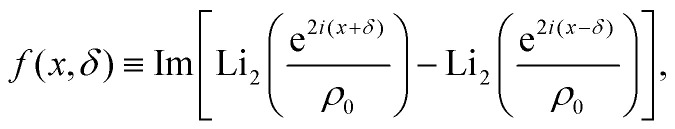
in which Li_2_ denotes the di-logarithm and *ρ*_0_ > 1 is a dimensionless parameter that smooths the potential, driving it away from divergent behavior. In ref. 53, this parameter corresponds to the distance between the colloid center and a companion topological −1/2 defect normalized by the colloid radius. Large *ρ*_0_ leads to a reduction of the details of the potential, which becomes independent of *δ* and fixes its width. As such, we fix *ρ*_0_ = 1.2 throughout.

**Fig. 2:**
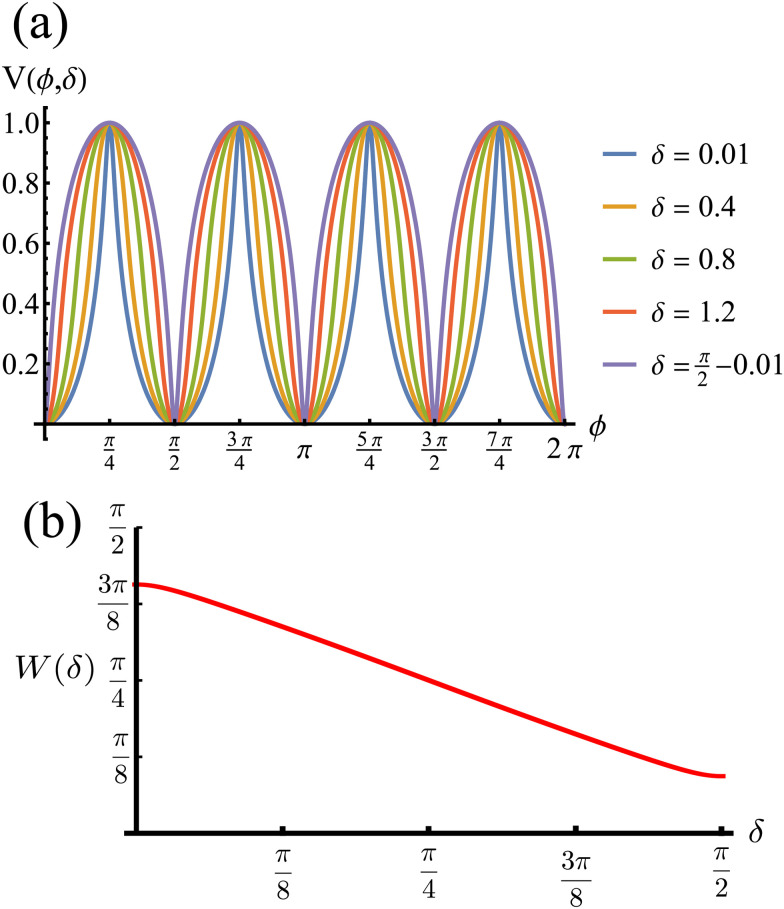
(a) Rotational potential as a function of *ϕ* for different values of *δ*. The potential wells become narrower as *δ* increases. (b) Width of the potential wells *W* as a function of *δ*. The width is monotonically decreasing, behaving almost linearly. Because *ρ*_0_ > 1 (see eqn (3)), the width does not reach zero at any value of *δ*, thus contributing to the numerical stability of our solutions for the Langevin equation. *V*, *W* and *δ* are all dimensionless.

The potential achieves its minimal value of *V* = 0 at *ϕ* = 0, π/2, π, and 3π/2 (Fig. 2(a)). Similarly, its maxima occur at *ϕ* = π/4, 3π/4, 5π/4 and 7π/4 with a value of *V* = 1. As such, by construction, the height of the potential barrier is Δ*V* = 1, regardless of the value of *δ*, with the angular mobility *Γ* controlling the effective barrier height. However, the width of the potential wells *W*, defined as the angular difference between midpoints of the potential (*i.e.*, *V* = 0.5), decreases monotonically with *δ* (Fig. 2(b)). The derivatives of the potential have compact closed expressions5
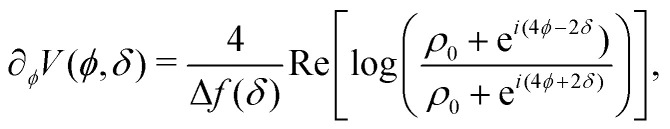
6
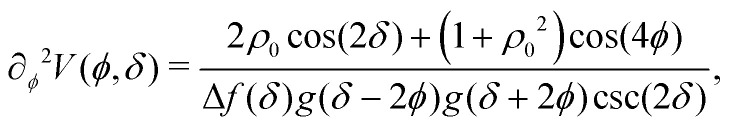
in which we have defined Δ*f*(*δ*) = *f*(π,*δ*) − *f*(π/2,*δ*), and 
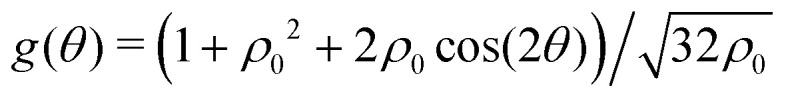
.

While eqn (1) describes the self-propulsion of the colloid, eqn (2) describes the particle's steering dynamics, with the particle's polar angle wiggling around one of *V*'s minima driven by noise, whose strength we quantify by *D*_r_. We begin by simply integrating eqn (1) and (2) numerically for different values of *δ* using *Γ*/*D*_r_ = 7.0. For low values of *δ* (*i.e.*, broader potential wells), the particles' orientations can explore a larger neighborhood around their minima. Thus we observe trajectories with a wider spread around the coordinate axes. However, these deviations are short lived as the angular potential quickly pushes them back to the minimum. This leads to horizontal or vertical trajectories when viewed on long-length and -time scales. As *δ* is increased, the wells narrow, and the orientation becomes increasingly trapped near the potential minima of the potential. As a result, the particle follows well-collimated trajectories: straight lines with sharp right turns (Fig. 1), thus giving the impression of the particle moving on a well-defined lattice. The trajectory can be thought of as the configuration of a lattice polymer, or their continuum limit, with rigid and fixed right-angled bends.^55^ As such, the interplay between the angular potential and the angular noise results in two effects on the statistical behavior of the particle's dynamics: one at short-time scales and the other at long-time scales.

### 2.1 Characteristic timescales

**Fig. 3:**
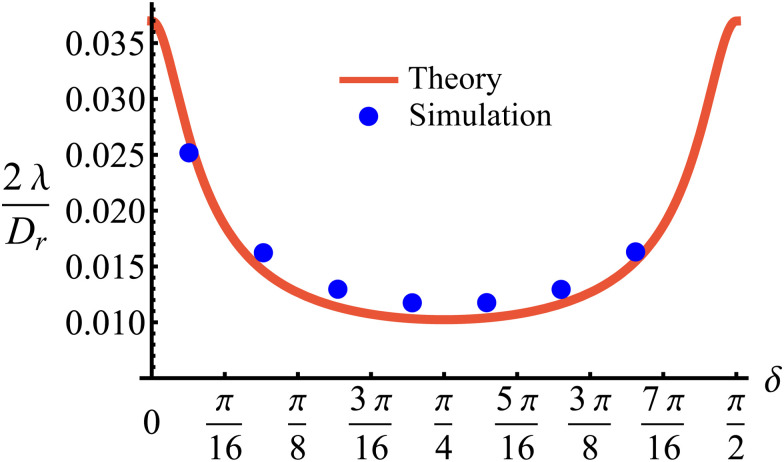
Total tumble-turn rate (2*λ*) as a function of the potential well's width *δ*. The red curve corresponds to the theoretical approximation (eqn (8)), whereas the blue dots are obtained by estimating the tumble-turn rate from the tangent–tangent correlation function (*i.e.*, 〈cos[*ϕ*(*t*) − *ϕ*(*t* + *τ*)]〉).

At short-time scales, the orientation of solitary particles diffuses around the minima of the potential, leading to small deflections of the particle's velocity with respect to the system's axes. Depending on the width of the potential and the strength of the noise, this results in a less-collimated/broader distribution along the axes, with the particles taking longer to travel a fixed distance along the preferred axis. Over longer time scales, however, the noise induces a finite probability for the polar angle *ϕ* to jump to a neighboring minimum. Any such jump amounts to a sharp right-angle turn, which amounts to a tumble-turn event in terms of being a sudden change to a previously persistent direction of motion. The timing between tumble-turn events corresponds to the mean time required to overcome the potential barrier *τ*. As such, it will depend exponentially on the effective height of the barrier *Γ*, the strength of the noise *D*_r_, and the curvature near the extrema. This is approximated using Krammer's formula7
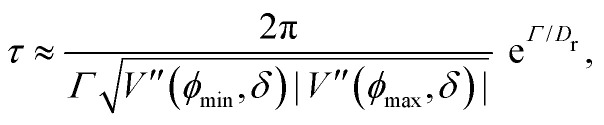
in which *ϕ*_min_ and *ϕ*_max_ denote neighboring minima and maxima, respectively. As such, the tumble-turn rate *λ* = 1/*τ* is found to be8
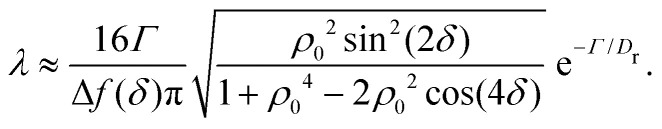
This tumble-turn rate only accounts for one neighboring minimum. Since each minimum is surrounded by two neighbors, the total tumble-turn rate is 2*λ*. Per eqn (8), the only dependence of the turn rate on the shape of the well is through *δ*. The tumble-turn rate increases substantially as *δ* approaches *δ* = 0 and π/2 (Fig. 3) because the curvature of *V*(*ϕ*,*δ*) at its minima (maxima) increases substantially as *δ* → π/2 (*δ* → 0).

In addition to the theoretical prediction eqn (8), Fig. 3 also shows numerical estimations for *λ*, measured from the simulations as the characteristic time of the tangent–tangent correlation function, *i.e.*, 〈cos[*ϕ*(*t*) − *ϕ*(*t* + *τ*)]〉. Both estimations agree with each other, with the theoretical curve having a slight underestimation for intermediate values of *δ*. Nevertheless, the total tumble-turn rate provides a natural time scale *T* = (2*λ*)^−1^ and length scale ℓ = *v*_0_/(2*λ*).

### 2.2 Long-time behavior

**Fig. 4:**
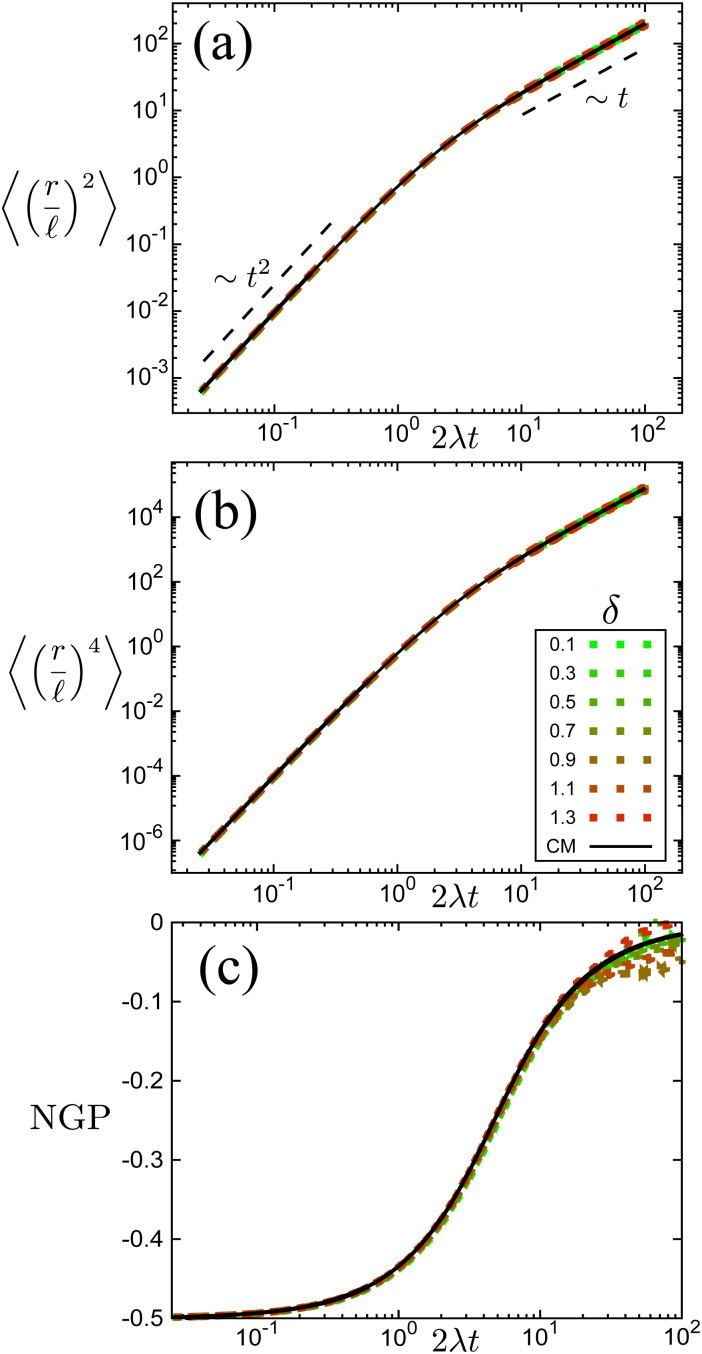
Normalized moments and non-Gaussian parameter NGP from simulations of both the Langevin equation, eqn (1) and (2), for various potential widths *δ* at *Γ*/*D*_r_ = 7 (dashed lines), and the continuum master equation, eqn (21) (solid lines, labeled CM). (a) Mean squared displacement (MSD), (b) fourth moment, and (c) the non-Gaussian parameter (NGP).

The time scale *T* defines what constitutes short and long times, as it controls in its entirety the shift from propulsive to diffusive behavior for solitary run-and-tumble-turn particles, as it can be observed by analyzing the moments of the particles' trajectories. Using the particles' position over time, and assuming the particles start from the origin, we compute the displacement's second (MSD = 〈*r*^2^〉) and fourth (〈*r*^4^〉) moments, as well as the non-Gaussian parameter (NGP = 〈*r*^4^〉/(2〈*r*^2^〉) − 1). The non-Gaussian parameter measures the extent to which the fourth moment deviates from the value of a Gaussian with the same second moment. Fig. 4 depicts these for various values of *δ* after rescaling them using *T* and ℓ as our units of time and length, respectively. The resulting collapse of all curves into one for all these three measurements, Fig. 4(a)–(c), clearly shows that the long-time behavior of the dynamics is controlled entirely by *T*, and therefore, by the tumble-turn rate *λ*.

The MSD (Fig. 4(a)) shows a clear propulsive regime at *t* ≪ *T* and a transition to a propulsive regime for *t* ≫ *T*. The propulsive dominated dynamics at small *t* is also revealed by the negative sign of the NGP (Fig. 4(c)), which for a purely ballistic particle, has a value of −0.5. As particles begin to turn and enter a diffusive regime, their distribution becomes increasingly more isotropic, thus increasing the NGP. At very large *t*, the dynamics are essentially diffusive, thus leading to a normal distribution, as highlighted by the NGP approaching zero. Similar results were observed in ref. 35 and 36 for run-and-tumble processes with anisotropic kernels: when different turns relative to the current direction of motion of the particles have different weights assigned to them. In particular, the temporal mismatch between the linear regime of the MSD and vanishing NGP seems to be an essential feature of run-and-tumble-like motion.^35^

### 2.3 Short-time propulsive behavior

In addition to the turning dynamics that govern long-time behavior, the solitary run-and-tumble-turn particle also exhibits short-time wiggling around the minima of the angular potential. Even though this short time can broaden the particle's spatial distribution, it preserves propulsive dynamics. This is because a particle never travels back in the opposite direction, maintaining, on average, a constant direction of motion.

This hand-waving argument can be put into more formal grounds by studying the moments of the spatial probability distribution associated with eqn (1) and (2). Since we are currently interested in studying the effects of noise near the minima and do not yet consider barrier jumping dynamics, a coarse approximation to the potential in eqn (2) will suffice and, in what follows, a harmonic approximation is employed, *i.e.*, *V*(*ϕ*,*δ*) ≈ *kϕ*^2^/2, in which *k* = ∂_*θ*_^2^*V*(*ϕ* = 0, *δ*). With this approximation, eqn (2) becomes the Langevin equation of an Ornstein–Uhlenbeck process, whose associated Fokker–Planck equation has a well-known solution^56^9
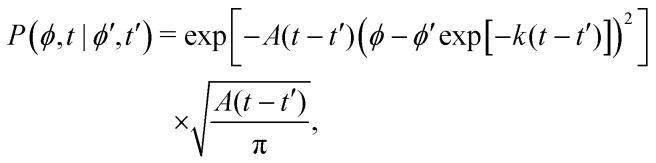
in which *A*(*t*) ≡ *k*(*D*_r_[1 − exp(−2*kt*)])^−1^. Since the solution to eqn (1) for a particle starting from the origin is10
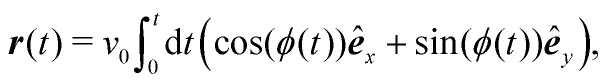
the MSD for a particle with its origin at its initial position is11
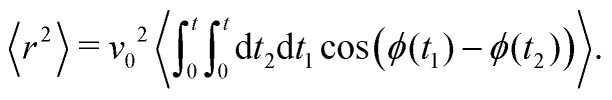
Computing 〈*r*^2^〉 requires the probability of finding at time *t*_2_ an orientation *ϕ*(*t*_2_) = *ϕ*_2_ knowing that at *t*_1_ it had an orientation *ϕ*(*t*_1_) = *ϕ*_1_. Namely, we assume without loss of generality that *t*_2_ ≥ *t*_1_. Using eqn (9) with *A* = *A*(*t*_1_) and *B* = *A*(*t*_2_ − *t*_1_) and *α* ≡ exp(−*k*(*t*_2_ − *t*_1_)) this probability is12
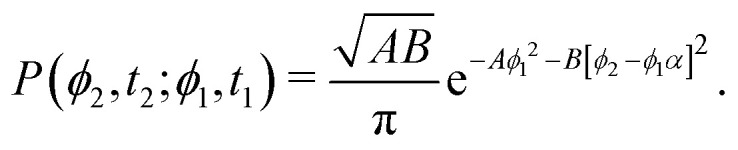
Thus, one must compute13
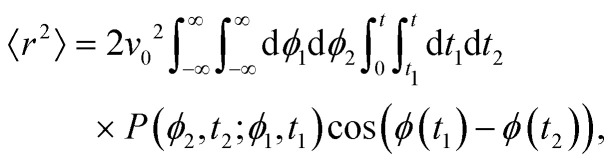
in which the factor 2 accounts for the opposite time order (*i.e.*, *t*_1_ ≥ *t*_2_). Performing the integrals over *ϕ*_1_ and *ϕ*_2_ leads to14
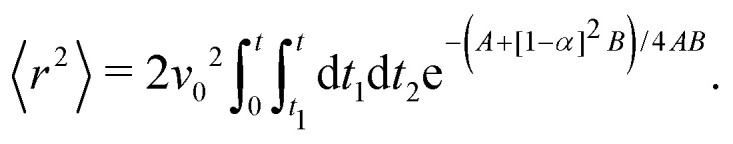
Changing the variables of integration *t*_1_ and *t*_2_ to *u* = *k*(*t*_1_ + *t*_2_) and *v* = *k*(*t*_2_ − *t*_1_), the integral becomes15
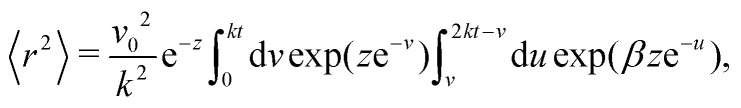
in which *z* = *D*_r_/*k*, and *β* = cosh(*v*) − 1. We now perform the inner integral and arrive at16
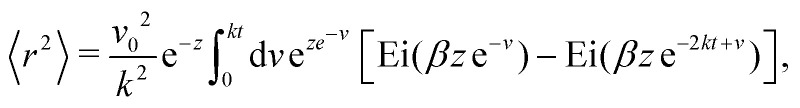
in which Ei denotes the exponential integral function.

To proceed forward, we recognize that for the case at hand (*i.e.*, for high enough barriers), fluctuations are expected to be smaller than π/2 (Fig. 2(a)), such that the strength of the noise is much smaller than the force derived from the potential (*i.e.*, *k*). Namely, the relaxation time to the minima of the potential 1/*k* is much shorter than the persistence time of the noise 1/*D*_r_. With this, we have that *z* = *D*_r_/*k* ≪ 1, and the arguments inside the exponential integral functions are much smaller than 1, allowing the expansion: Ei(*x*) ≈ *γ*_E_ + *x*(4 + *x*)/4 + ln(*x*) (for *x* ≪ 1), in which *γ*_E_ denotes Euler's constant. Furthermore, after the expansion, the integrand can be well approximated by its linear expansion around *kt*/2. With the above approximations, the last integral in eqn (16) can be evaluated. The result is a lengthy expression; however, as we are interested in the long-term consequences of the fluctuating movement of the orientation around the minima, it makes sense to consider the large *t* limit, *i.e.*, *t* → ∞. In this limit, the integral can be largely simplified to17
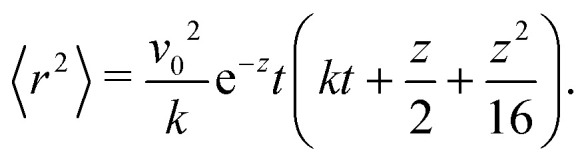


Since *z* ≪ 1, this implies that, as *t*→∞

18 〈*r*^2^〉 ≈ *v*_0_^2^e^−*z*^*t*^2^ = (*v*_0_e^−*D*_r_/(2*k*)^t)^2^.

In other words, even at long times within the short-time regime, the MSD grows propulsively as ∼*t*^2^ with an effective velocity

19 *v*_eff_ = *v*_0_ exp(−*D*_r_/2*k*) ≤ *v*_0_.

**Fig. 5:**
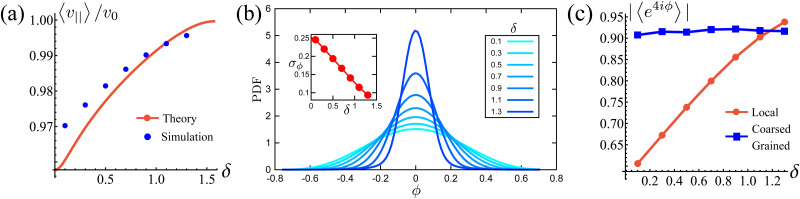
(a) Normalized effective velocity along the preferred axes 〈*v*_‖_〉. The red curve is the theoretical prediction from eqn (19). Blue dots are measured directly from the simulation. (b) Distribution of the instantaneous polarization angle *ϕ*, translated to the interval [−*ϕ*/4,*ϕ*/4], as function of potential width *δ*. Inset: Standard deviations as functions of *δ*. (c) Measure of squarishness using instantaneous *ϕ* (red curve) and *ϕ* coarse grained over *T* (blue curve).

In contrast to the MSDs reported in ref. 6 and 57, which exhibit the traditional transition from a propulsive ∼*t*^2^ short-time behavior to a diffusive ∼*t* long-time behavior, the MSD for run-and-tumble-turn dynamics described by eqn (18) is in a perpetual *t*^2^ regime. The MSD never shifts to ∼*t* behavior. The renormalization of the velocity occurs because the particle's velocity deviates further from its preferred direction as the noise increases, resulting in the particle translating more slowly along the axis. We can measure such an effect in our simulations by averaging the component of the instantaneous velocities parallel to the instantaneous preferred traveling axis, 〈*v*_‖_〉 = *v*_eff_. Fig. 5(a) shows this estimate for different *δ*'s and compares it with the theoretical prediction from eqn (19). The estimations agree on the magnitude and in the monotonicity of *v*_eff_ with *δ*, with the theoretical curve under-predicting simulations for low *δ* because the steering potential (eqn (3)) has been approximated as harmonic.

It is natural to ask how much the width of the potential affects this deviation from the preferred direction and how, in turn, this affects the squarishness of the particle's trajectories. To measure the directional deviations, we sample the distribution of instantaneous polarization angles *ϕ* (Fig. 5(b)). This only shows angles between −π/4 and π/4, as, akin to a first Brouillian zone, we have translated values of *ϕ* centered around other minima. It is clear that the shape of the distributions becomes increasingly sharper as *δ* increases, which is quantified by their linearly decreasing standard deviation, shown in the inset. This agrees with our notion of narrower potentials being more collimated.

It is striking, however, how broad the distribution appears to be at small *δ* (Fig. 5(b)), as this would suggest that the trajectories are not very square-like. The answer to this apparent contradiction is that it is a matter of scales. Inspired by the bond orientational order parameter,^58^ we define the squarishness of a trajectory as *S* ≡ |〈exp(4*iϕ*)〉|. This directly measures the four-fold symmetry of our particles' trajectories: for isotropic trajectories *S* ≈ 0, whereas for particles traveling mainly along lines intersecting at a π/2 angle *S* ≈ 1. The red line in Fig. 5(c) depicts *S*, measured using the instantaneous values of *ϕ*, as a function of *δ*. In addition to showing that *S* increases strongly and monotonically with *δ*, this figure shows that *S* has intermediate values at small *δ* that are not consistent with squared trajectories, but also incompatible with disordered trajectories. These values reflect that at small *δ* the particles have noisy trajectories that mostly travel along some preferred axes. As such, the trajectory will look very noisy and not very ordered when looking over small distances and short-time scales. However, when viewed from afar, these fluctuations should cancel out, leaving behind only the directions of the preferred axes and, therefore, a high value of *S*.

Indeed, if instead of using instantaneous values of *ϕ* to compute *S* we use coarse-grained values obtained by computing running averages of *ϕ* for each trajectory over the tumble-turning time scale *T* = (2*λ*)^−1^, we see that the coarse-grained *S* is independent of *δ* and it has a high value, of the order of 0.9, as depicted by the blue curve in Fig. 5(c). Moreover, this implies that the trajectories are always squared when looked at from the length scale of the particle's persistent length, with the deviations only affecting the short-length-scale dynamics, which agrees with our qualitative intuition from Fig. 1.

In summary, there is a distinct separation of scales: (i) the random walk about the potential controls the short-time behavior, renormalizing the velocity, and (ii) at long-time scales, overcoming of the potential barrier and tumble-turning controls the dynamics, transitioning the behavior from propulsive to diffusive. Moreover, when considered from the perspective of the persistent length, the dynamics and trajectories of these colloids become indistinguishable from one another, regardless of the width of the potential. Thus, a continuum theory that captures just the essentials of the run-and-tumble-turn dynamics, namely the right-angled turns, and disregards the details encompassed by the width of the potential is likely to capture most of the system's dynamics.

## 3 Master equation for a single particle

Through the rotational potential, solitary run-and-tumble-turn particles interact with the environment's inherent coordinate system, which drives particle dynamics to lie approximately parallel to one of the coordinate axes. This section presents a continuum master equation for these particles. For the sake of simplicity, we assume the limit of very narrow potential wells, *i.e.*, *δ* → π/2 (Fig. 2(a)), so the small variations in the particle's propulsion are negligible when its orientation deviates from the minima of the potential. As such, the particle can only move in four directions: rightwards, upwards, leftwards, and downwards. As a consequence of assuming only sharp trajectories, the stochastic orientational dynamics described in the previous section result in the particle experiencing random sharp π/2 turn. Since, in the Langevin description, the jumps are restricted to neighboring potential wells, the particle cannot directly do a u-turn, reversing its direction. As such, the entirety of the hopping dynamics is encompassed by the tumble-turn rate *λ* (eqn (8)).

With these simplifications in mind, the particles' self-propulsion dynamics reduce to a Poisson random walk, which can be described as probability densities. As the colloid can only move in four directions, there are four probability densities: *P*_→_(**r**,*t*), *P*_↑_(**r**,*t*), *P*_←_(**r**,*t*), *P*_↓_(**r**,*t*), which denote the probability of having a colloid in a neighborhood *d*^2^*r* of position ***r*** at time *t* traveling right, up, left or down, respectively. Since the particle always travels at the same speed *v*_0_ and turns with probability rate *λ*20
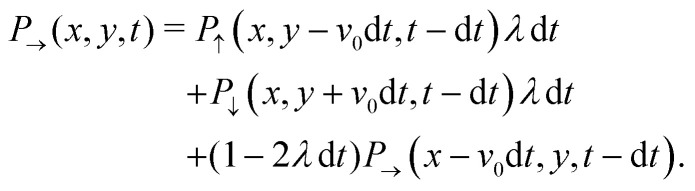
This simply states that the probability of finding a particle at position ***r*** at time *t* traveling to the right is the sum of the probabilities of having the same particle at an earlier time *t* − d*t*, with *λ*d*t* ≪ 1, traveling either up or down and turning, or traveling to the right and not turning. There are similar expressions for the remaining three probability densities, which become exact in the limit d*t* → 0. Expanding these expressions in d*t* and keeping only terms up to first order produces the dynamical equation for the probability spinor ***P*** = (*P*_→_, *P*_←_, *P*_↑_, *P*_↓_). Taking *T* = 1/(2*λ*) and ℓ = *v*_0_ / (2*λ*) as the unit of time and length, respectively, this dynamical equation takes the following dimensionless form

21 ∂_*t*_***P*** = −***H***_D_***P***,

in which the dynamical matrix is22
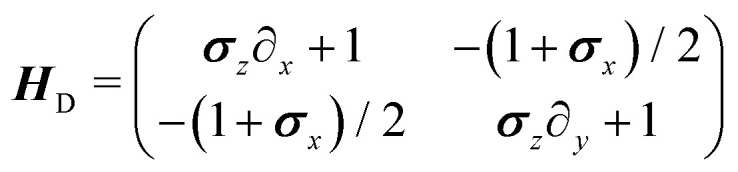
for the Pauli matrices ***σ***_*z*_ and ***σ***_*x*_. Notice that the master equation, eqn (21), corresponds to the continuum limit in both time and space of a particle model in a square lattice.

**Fig. 6:**
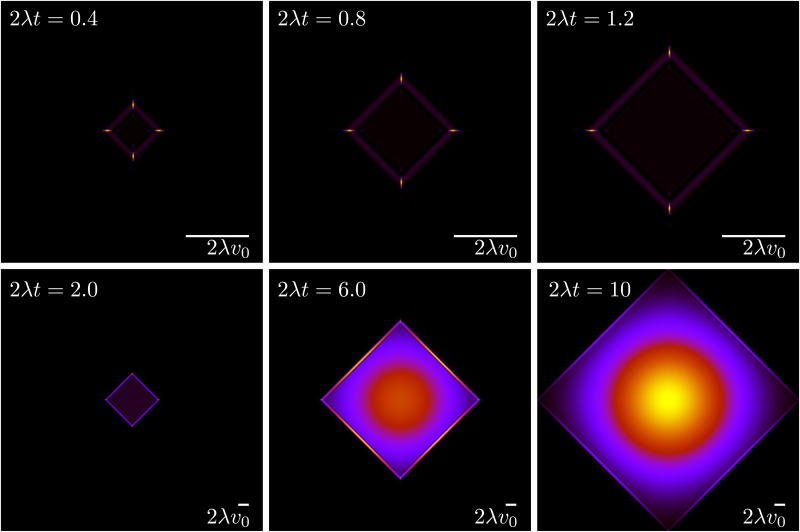
Time evolution of the total probability *ρ* = *P*_→_ + *P*_↑_ + *P*_←_ + *P*_↓_ (eqn (21)), of an initially well-localized distribution. Black denotes 0 probability density, whereas yellow denotes high probability density. The upper row (short times) has a different spatial scale than the lower row (long times).

The evolution for the total probability *ρ* = *P*_→_ + *P*_↑_ + *P*_←_ + *P*_↓_ under eqn (21), with a localized initial distribution without any preferred axis, is depicted in Fig. 6. At short times (upper row of Fig. 6), when propulsive dynamics dominate, the distribution has a marked 4-fold symmetry, with 4 probability peaks traveling along the axes with speed *v*_0_. As time reaches the characteristic time *T*, most particles have turned once, forming diagonal fronts that join the probability peaks. At larger times (lower row of Fig. 6), particles have had enough time to turn at least twice and do u-turns, thus returning to their initial position, incrementing the relative value of the probability distribution at the origin. At the same time, most particles have turned at least once, moving the maxima of the distribution out of the traveling peaks into the center of the diagonals. Finally, at long times, the dynamics are diffusive, leading to a normal distribution at an increasingly larger distance near the origin. At length scales comparable with *v*_0_*t*, the probability distribution still has a 4-fold symmetry due to the diagonal front of particles that have turned at most once.

**Fig. 7:**
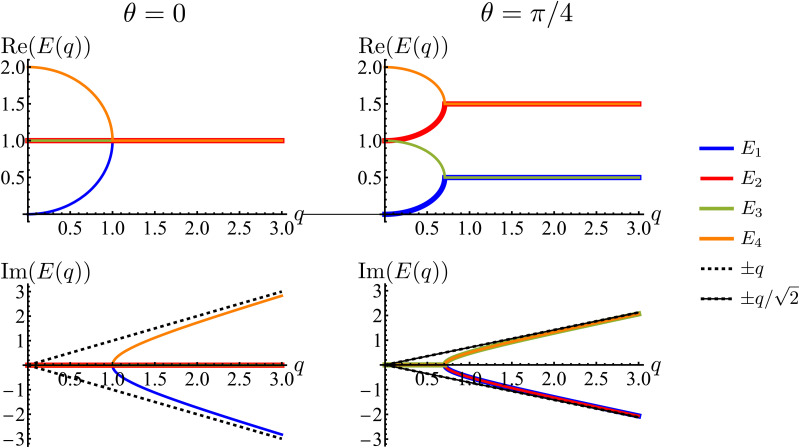
Real and imaginary parts of the eigenvalues of the dynamical matrix, eqn (23), as functions of the radial wave-number *q* and its polar angle *θ*.

**Fig. 8:**
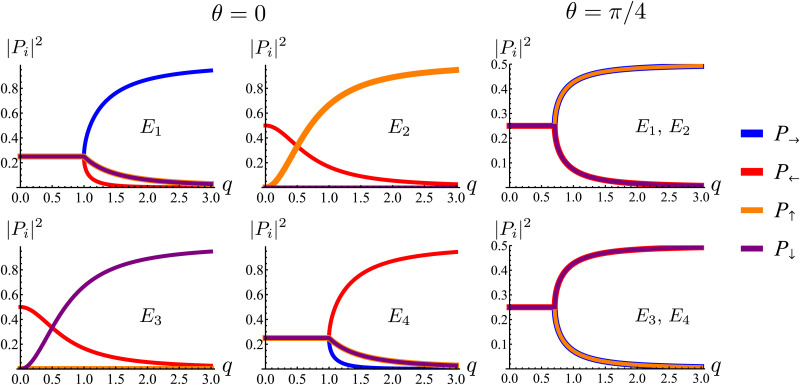
Square of the absolute value of the components of the eigenmodes of the dynamical matrix in eqn (22) as functions of the radial wave-number *q* and its polar angle *θ*.

Much of the behavior of eqn (21) can be inferred from analyzing the spectrum of ***H***_D_. *Via* Fourier transformation, the eigenvalues of ***H***_D_ are23
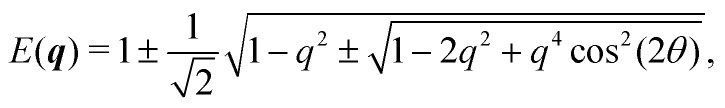
in which *q* = |***q***| and *θ* = atan2(*q*_*y*_,*q*_*x*_). These are depicted in Fig. 7. The components of the associated eigenvectors are shown in Fig. 8.

First, the spectrum is explicitly anisotropic, as it has an explicit dependence on *θ*, which is a consequence of the system's underlying four-fold symmetry. The noisy term associated with diffusive behavior enters as a second derivative in Fokker–Planck equations and so diffusive modes are associated with a purely real, quadratic spectrum ∼*q*^2^. In contrast, traveling modes, which usually arise due to drift currents, are associated with the imaginary parts of spectra. Let {*E*_i_}^4^_i=1_ denote the four eigenvalues of eqn (23). At long wavelengths (*i.e.*, small *q*), the system is completely diffusive as the spectrum is purely real and 
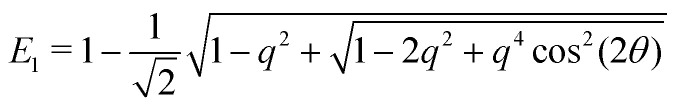
, which is the only hydrodynamical mode (the only one that survives at long time scales due to having the smallest real part), goes to zero as *E*_1_ ∼ *O*(*q*^2^) for *q* ≪ 1 (Fig. 7). Furthermore, at long wavelengths, all components of the eigenvector associated to *E*_1_, and hence all propagating directions, have the same weight (top-left and top-right panels of Fig. 8). Thus, the dynamics become isotropic at long times.

More interesting is what happens at short length scales (large *q*)—along the *x* axis (*θ* = 0), each mode can be identified with one direction of propagation, as they only have one non-zero component (Fig. 8). Moreover, although all modes decay at the same rate, only two acquire an imaginary part and can therefore propagate (left panels of Fig. 7). These are precisely the modes associated with movement along the horizontal axis: *E*_1_ and *E*_4_ (top-left and bottom-right panels of the *θ* = 0 section of Fig. 8). Furthermore, the different sign in the imaginary part of these modes ensures that *E*_1_, associated with *P*_→_ has modes traveling to the right; whereas *E*_4_, associated with *P*_←_, has modes traveling to the left. Since the system has a four-fold symmetry, the same picture is evident along the vertical axis, but with propagation in the upward or downward directions.

Along the diagonal *θ* = π/4, the components do not fully decouple. Instead, pairs of components have the same weight, the first pair being *P*_→_ and *P*_↑_, and the second *P*_←_ and *P*_↓_ (Fig. 8, right column). These pairs also share the same sign for the imaginary part of their spectrum (Fig. 7, bottom right), allowing the modes to travel toward the first and third quadrants, respectively. Regarding how fast these modes travel, it is seen from the asymptotic lines in the bottom row of Fig. 7 that they do so at a speed of *v*_0_, the natural unit of velocity, which is equally distributed along two components along the diagonal. Combining these results, for a well-localized initial condition, the system initially exhibits propulsive behavior as the distribution travels in the direction associated with each component, propelled by the traveling modes at short wavelengths. However, as particles start to turn, the modes begin to decay, with short wavelengths decaying faster than long ones. In the limit *t* ≫ *T*, this discrepancy in decay rates results in only one isotropic mode surviving, with purely diffusive dynamics.

Finally, comparing simulations of this continuum model, eqn (21) with the statistics derived from agent simulations (eqn (1) and (2)), in particular, the MSD, fourth moment and NGP (Fig. 4) reveals that the continuum model captures the essential features as the curves collapse. This reinforces the idea that when looking under the scale of ℓ and *T*, the dynamics are independent of the underlying details of the microscopic model and thus well described by the simplified continuum description. Although this section has focused on the most anisotropic case of sudden 90° left or right turns, the theory can be extended to consider an arbitrary number of directions (Appendix B).

## 4 The diffusive regime: long-wavelength theory

We have demonstrated two properties of the long-time dynamics of solitary run-and-tumble-turn particles: (i) diffusivity (Fig. 4(a)) and (ii) isotropy (Fig. 4(b)). However, it is not necessarily true that both properties emerge simultaneously. For example, intermediate-time dynamics may exhibit anisotropic diffusion or may be better described as isotropic propulsion. This section develops a hydrodynamic theory of the model to study the anisotropy of the diffusive regime and the approach to isotropy.

The eigenvalue analysis of the spinor dynamics (eqn (23)) revealed the presence of a hydrodynamic mode, suggesting this model supports a simplified long wavelength theory. To obtain it, notice that the particle density is *ρ* = *P*_→_ + *P*_←_ + *P*_↑_ + *P*_↓_, the current is ***j*** = (*P*_→_ − *P*_←_)***ê***_*x*_ + (*P*_↑_ − *P*_↓_)***ê***_*y*_, and the degree of nematic ordering is described by *χ* = (*P*_→_ + *P*_←_) − (*P*_↑_ + *P*_↓_), which is positive if it is more likely for the particle to propagate along the horizontal axis and negative if it is more likely to propagate along the vertical axis. With these definitions, the spinor dynamics (eqn (21)) become

24 ∂_*t*_*ρ* + **∇** · ***j*** = 0,

25
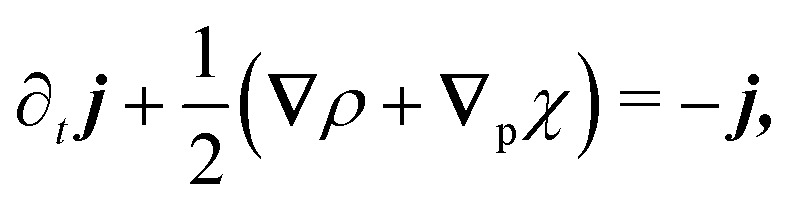

26 ∂_*t*_*χ* + **∇**_p_ · ***j*** = −2*χ*,

in which **∇**_p_ ≡ (∂_*x*_, − ∂_*y*_). Since the system does not possess particle–particle interactions that may lead to polar or nematic order, and *ρ* is the only conserved quantity, *ρ* is the only possible hydrodynamical variable. As such, ***j*** and *χ* act as fast variables, and their time derivatives are negligible.^59^ Recursively substituting eqn (25) and (26) into eqn (24) produces the following modified diffusion equation27
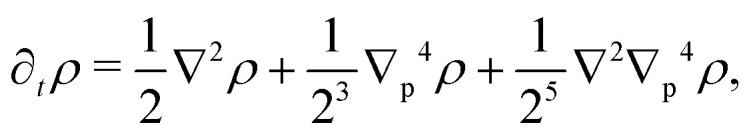
in which the differential operator ∇_p_^2^ ≡ ∂_*x*_^2^ − ∂_*y*_^2^ serves as the source of anisotropy in the model. Through ∇_p_, the anisotropy appears on gradients of fourth order or higher, thus ensuring that any anisotropy in the system will be short lived. Although one ideally looks to truncate derivatives in the smallest possible order, it is necessary to go to the sixth order in this case. While truncating at the second order will just yield the diffusion equation, truncating at the fourth order will result in instabilities arising at short wavelengths. As such, the sixth-order derivative is necessary to stabilize the equation at short wavelengths.

**Fig. 9:**
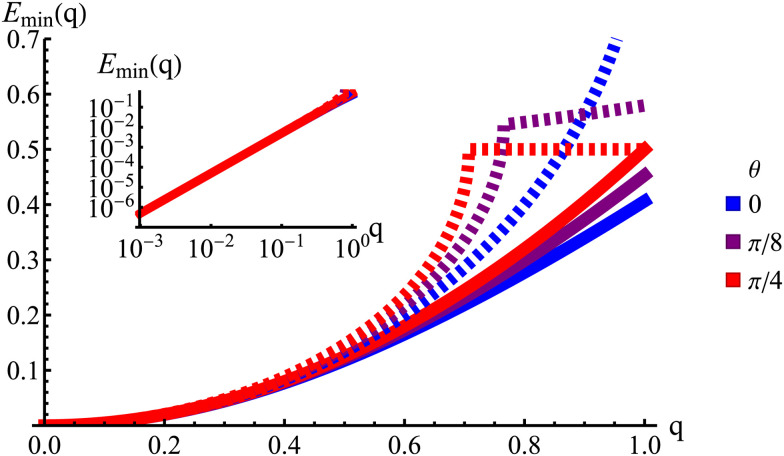
The lowest eigenvalue of eqn (23) (dashed lines) and eqn (29) (solid lines) for different angles (blue: *θ* = 0, purple: *θ* = π/8 and red: *θ* = π/4). Inset: Log–log scale, demonstrating that the curves collapse at small wavenumbers *q*, showing that the theory is accurate at small wavenumbers.

In Fourier space, the spectrum of eqn (27) is given by28
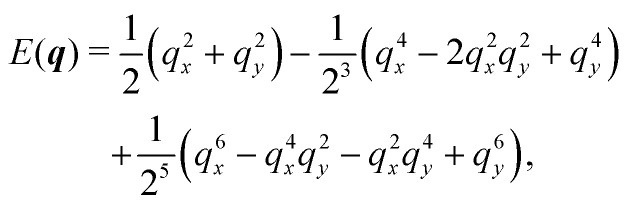
or, alternatively, in polar form (*q*_*x*_ = *q* cos(*θ*), *q*_*y*_ = *q* sin(*θ*))29
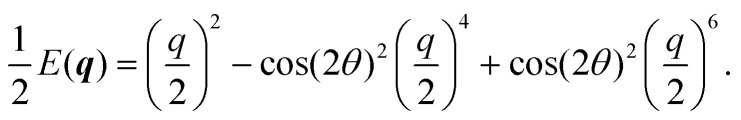
Because of its direct dependence on *θ*, *E*(***q***) is explicitly anisotropic. To compare the reduced hydrodynamic theory with the more detailed continuum theory, we compare this spectrum with the lowest eigenvalue in eqn (23) (Fig. 9).

The hydrodynamic theory matches the lowest eigenmodes but loses the details of the behavior at short-length scales. Consequently, the hydrodynamic theory is only valid when describing phenomenology occurring at length scales larger than 1, *i.e.*, *q* ≪ 1 (using ℓ as the unit of length). Furthermore, the theory is essentially diffusive as it loses all traveling modes. The fact that the model still presents anisotropic features while being fully diffusive reveals that the change to diffusive behavior precedes the change to the isotropic one.

Having derived the theory, it can now be used to study how long the anisotropic features last by describing the time evolution of an initial probability distribution *ρ*_0_ = *ρ*(*t* = 0)30
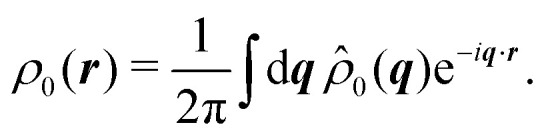
Because the theory is only valid at large length scales, we demand this initial distribution to have vanishingly small Fourier components at large wavenumbers, *i.e.*, *f̂*(***q***) ≈ 0 for *q* ≫ 1. For the sake of simplicity, we take *ρ*_0_ to be a normal distribution of variance *σ*^2^ ≥ 1. For *ρ*_0_(***r***) = exp(−*r*^2^/(2*σ*^2^))/(2π*σ*^2^), one finds *ρ̂*_0_(***q***) = exp(−*σ*^2^*q*^2^/2)/(2π), which is exponentially small at large *q* since *σ* ≥ 1. This condition prevents the initial distribution of displaying short wavelength features that the hydrodynamic theory cannot handle. The time evolution of the distribution is given by31
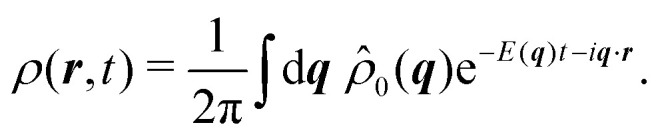
Because this integral is heavily suppressed for *q* > 1, the limit *q* ≪ 1 is always satisfied in the integrand. With this in mind, *q*^2^ ≫ *q*^4^, *q*^6^, which allows the approximation32
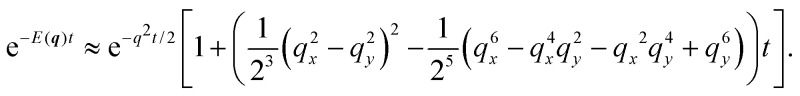
In turn, the integral over ***q*** in eqn (31) can be performed, yielding the time-dependent distribution33
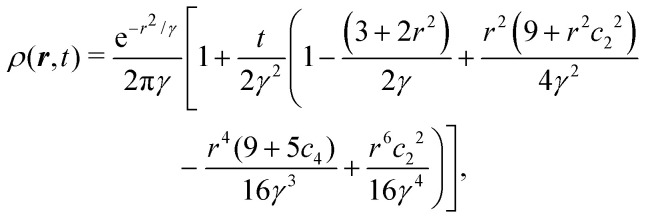
in which we let *γ* ≡ *σ*^2^ + *t* and *c*_*n*_ = cos(*nθ*) for brevity. As a first estimate of how anisotropic this distribution is, notice that for large *t*, the leading anisotropic term in eqn (33) decays in time as ∼cos^2^(2*θ*)/*t*^3^, which appears to suggest that any anisotropic feature would be short lived. However, this point of view is local and does not tell the entire story.

To study the distribution's global properties, consider its moments. The second moment represents the mean squared displacement

34 〈*r*^2^〉 = 2(*σ*^2^ + *t*),

which confirms the diffusivity of the model. Besides the *σ*^2^ term that originates on the variance of the initial distribution, we have that 〈*r*^2^〉 ∼ *t* at all times. Surprisingly the diffusion constant is *D* = 1/2, which is exactly the same result obtained by considering only the lowest gradients in eqn (24). As such, the anisotropic features of the dynamics do not seem to introduce anisotropic diffusion. Indeed, the diffusion coefficient along any line on the plane is the same: 1/4. This is because the local anisotropic dynamics do not make an explicit distinction between the Cartesian axes (see Appendix C). In summary, there are no anisotropic features at the level of the second moment.

However, this is not the case for the fourth moment

35 〈*r*^4^〉 = 8(*σ*^2^ + *t*)^2^ + 4*t*,

which is a different result than the one obtained considering only the lowest gradients (*i.e.*, 8(*σ*^2^ + *t*)^2^). This is due to the anisotropic features contributing to deviating the distribution from a Gaussian. The non-Gaussian parameter is36
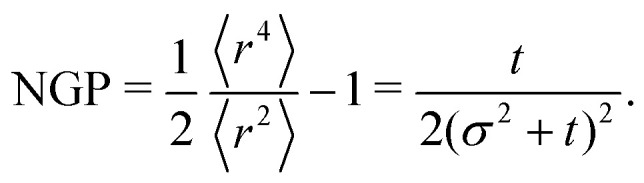
First, in this case NGP > 0, whereas for the master equation and agent simulations NGP<0 (Fig. 4(c)). This is reminiscent of repulsive active Brownian particles that can take positive or negative NGP depending on their dimensionless Péclet number of density,^60^ though our system always approaches NGP → 0 in the long time limit. This is because, unlike the agent's simulations, which are driven by propulsive forces, the anisotropy, in this case, is carried out diffusively. Second, NGP decays as *t*^−1^, indicating that the anisotropic features actually persist much longer than initially suggested by the *t*^−3^ decay observed in the distribution. Similar curves for the NGP, including the *t*^−1^ long-time limit, have been observed in a run-and-reverse with rotational diffusion model.^35^

**Fig. 10:**
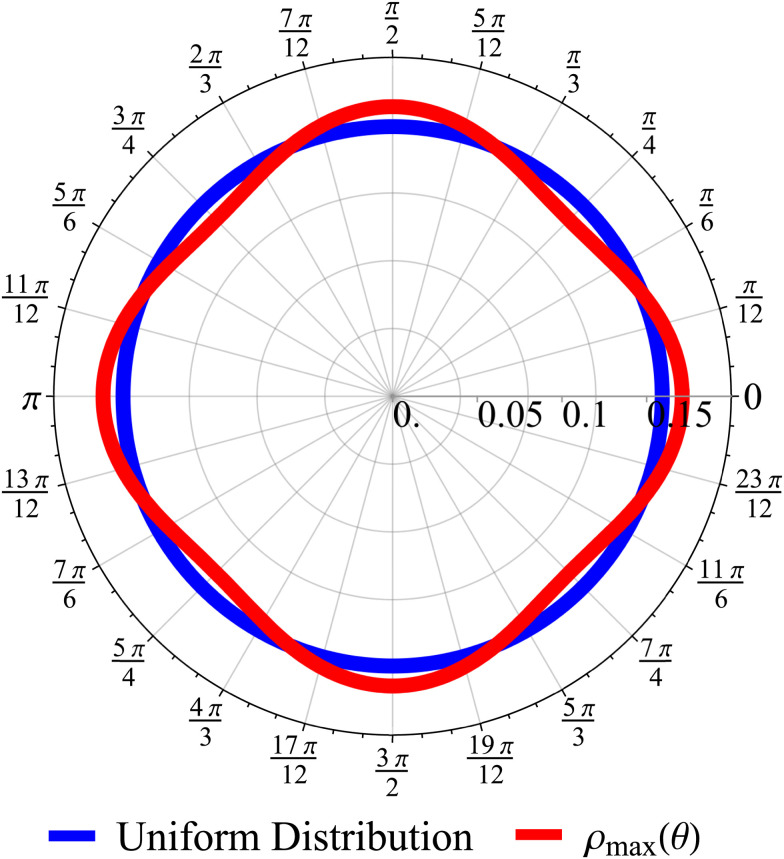
Angular distribution obtained from the long wavelength theory at the time of maximum anisotropicity *ρ*_max_(*θ*) (*t* = 2, *σ* = 1). The distribution can be noticeably anisotropic even in the diffusive regime. The maximum anisotropicity is not achieved at *t* = 0 (but rather *t* = 2) because the initial distribution is isotropic.

**Fig. 11:**
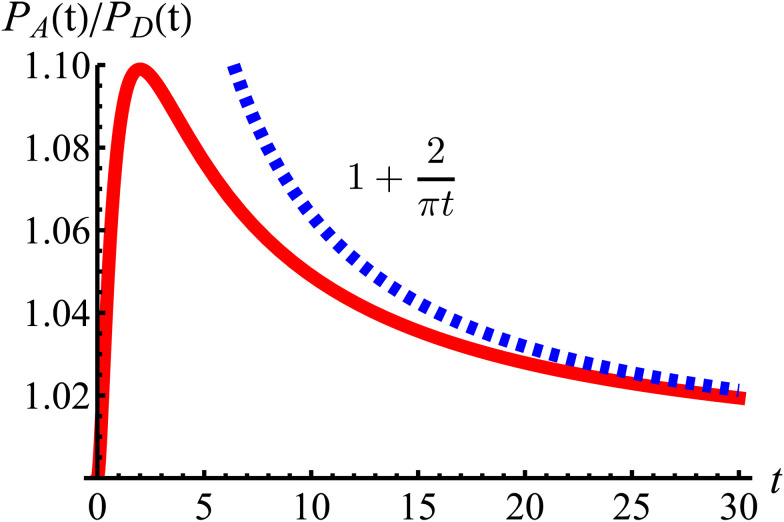
The odds of being closer to the axes *P*_A_ than closer to the diagonals *P*_D_ as a function of dimensionless time *t* (red curve). The blue dashed line is the long-time asymptotic behavior ∼1 + 2/(π*t*). The odds reach their maximum at *t* = 2 with an approximate value of 1.10. It decays as 1/*t*, keeping a value above 1.019 even after *t* = 30.

The *t*^−1^ decay of anisotropic features can also be corroborated by directly inspecting the angular distribution obtained by integrating the spatial distribution over the radial distance *r*, 
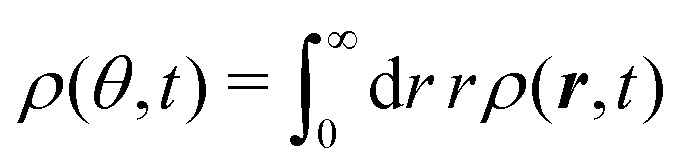
, which gives37
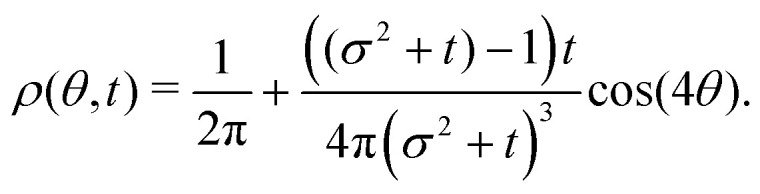
The non-monotonicity of *ρ*(*θ*,*t*) is a direct measure of the anisotropicity of the distribution (Fig. 10). This non-monotonicity is provided by the second term in eqn (37). Interestingly, this term decays in time as ∼*t*^−1^, just as the NGP, thus corroborating that this is the true rate of decay of the anisotropic features of the system, instead of *t*^−3^. Finally, the anisotropicity achieved by the system over time can be quantified by computing the probability of a particle being closer to the axes rather than the diagonal *P*_A_. Namely, four times the integral of *ρ*(*θ*,*t*) in the interval [−π/8,π/8]. Its reciprocal is the probability of being closer to the diagonals, *P*_D_. These are given by38
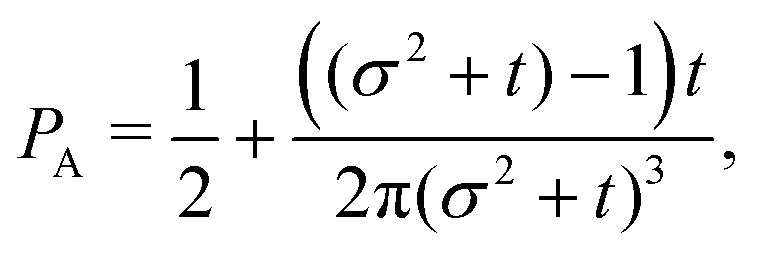
39
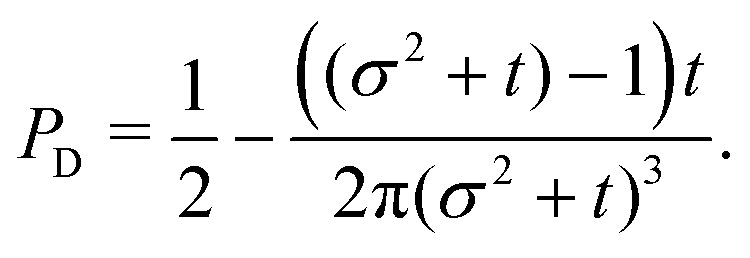
These show that *P*_A_ > *P*_D_ (since *σ*^2^ ≥ 1), reflecting that the particles prefer to travel parallel to the axes. The ratio between the two is depicted in Fig. 11 for *σ* = 1. This demonstrates that there is a temporal window on anisotropic dynamics. There is no difference at the beginning if the initial distribution is isotropic. However, anisotropy increases until the ratio reaches a maximum of 1.1 at *t* = 2 driven by the anisotropic dynamics in the model. The ratio decays as 1/*t*, maintaining a value of at least 1.019 even after *t* = 30.

## 5 Interacting particles: anisotropic flocking

Having studied the dynamics of solitary run-and-tumble-turn particles in detail, this section considers the case of several interacting run-and-tumble-turn particles and how the anisotropic features of single particles translate into collective dynamics. To do so, we return to the microscopic description and modify the particle's Langevin equations (eqn (1) and (2)) to incorporate forces representing short-range repulsive interactions and modeling particle–particle alignment40
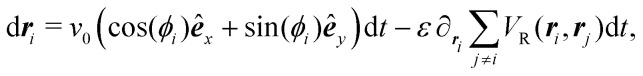
41
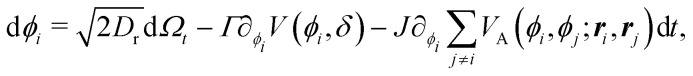
in which *V*_R_(***r***_*i*_, ***r***_*j*_) is the dimensionless potential associated with particle repulsion, *ε* > 0 sets the strength of the repulsion, *V*_A_(*ϕ*_*i*_, *ϕ*_*j*_; ***r***_*i*_,***r***_*j*_) is the dimensionless potential associated with particle alignment and *J* > 0 sets the strength of the alignment. Specifically, repulsion is modeled through a soft Herzian potential42
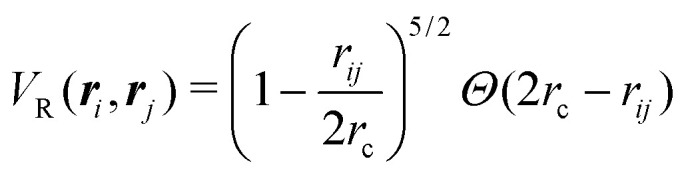
between particles *i* and *j*, in which *r*_c_ denotes the particles radius, *Θ*(*x*) is the Heaviside function and *r*_*ij*_ = |***r***_*i*_ − ***r***_*j*_|. This potential only acts when the particles are in contact. The alignment potential explicitly models particle–particle alignment as

43 *V*_A_(*ϕ*_*i*_,*ϕ*_*j*_;***r***_*i*_,***r***_*j*_) = −*Θ*(2*r*_A_ − *r*_*ij*_)cos(*ϕ*_*i*_ − *ϕ*_*j*_),

in which *r*_A_ is the range of the interaction; particles only align if the distance between them is smaller than 2*r*_A_. The total potential is the sum of eqn (3), (42) and (43). Having added these interactions to our model, we run simulations for *N* = 1000 particles using the same assumptions and parameters as previous sections in a box with periodic boundary conditions at a packing fraction of 0.5 (see Appendix A).

**Fig. 12:**
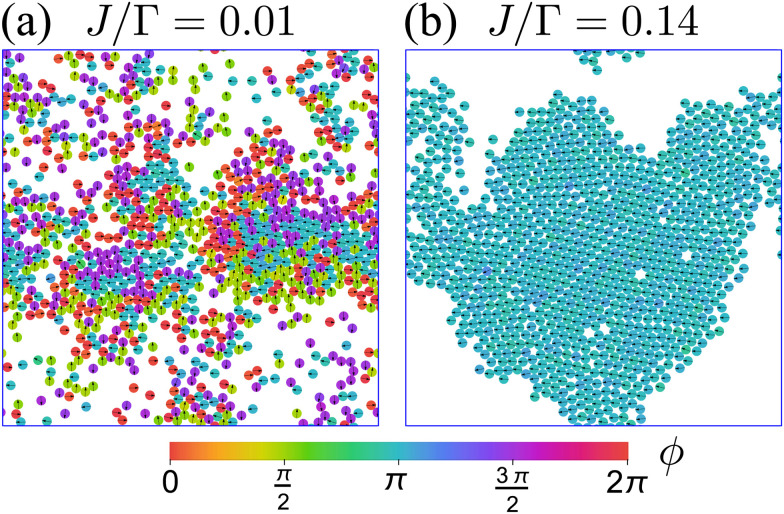
Snapshots representative of the behavior of a system of *N* = 1000, *δ* = 1.1, and packing fraction 0.5 at steady state for two different values of *J*. Particle color encodes particle orientation. (a) At small *J*, the system is in an isotropic state and presents MIPS. Animation included in Movie 1 (ESI†). (b) At larger values of *J*, the system transitions into an orientationally ordered/flocking state, in which a large fraction of particles (close to 1) acquire the same orientation. This orientation matches one of the four preferred directions set by the orientation potential (left, in this case). Animation included in Movie 2 (ESI†).

Fig. 12 displays the effects of these added interactions by displaying two snapshots of the system at two different values of *J*. When *J* is small compared to *Γ* (Fig. 12(a) and Movie 1, ESI†), the particle–particle aligning interaction *V*_A_ is insufficient to overcome the tumble-turn orientation potential *V*, and so particles individual orientations remain parallel along the coordinate axes. Furthermore, beyond small patches of polar order, there is no global orientation—the system is in an isotropic state. Nevertheless, the short-range repulsion has a strong effect on the system, driving it into a state of motility-induced phase separation (MIPS),^6^ which has been observed in other models with four-fold symmetry, such as active particles in a lattice.^61,62^ Since MIPS only depends on the particles possessing sufficient motility, it emerges even in the presence of anisotropic particle dynamics.

For larger *J*, the increased strength of the alignment interaction is high enough to drive the system into a flocking state/polar order (Fig. 12(b)). The system acquires global orientational order and a large fraction of particles point in the same direction. The polar order parameter used to quantify such a state is 
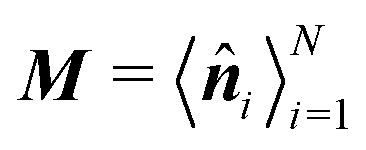
, in which ***n̂***_*i*_ = cos *ϕ*_*i*_***ê***_*x*_ + sin *ϕ*_*i*_***ê***_*y*_ is averaged over the particle ensemble. The scalar order parameter *S*_P_ = |***M***| quantifies the degree of polar order in the system, while *θ*_P_ = atan2(−*M*_*y*_,−*M*_*x*_) + π describes the global orientation, in which *M*_*x*_ and *M*_*y*_ denote the *x* and *y* components of ***M***, respectively.

Because particles are initialized at random positions and orientations, there is early clustering of particles. As the particles cluster, the alignment interaction becomes more significant, causing the run-and-tumble-turn particles to spontaneously acquire a collective direction of motion. The particles coalesce into a single system-spanning cluster, thus reinforcing the global orientation in a large fraction of the system's particles. Individual tumble-turn events are overwhelmed by the flocking alignment. Once all particles travel in the same direction, they do not collide with each other and are free to relax their repulsive interactions, leading to minimal overlap and high structural order.

**Fig. 13:**
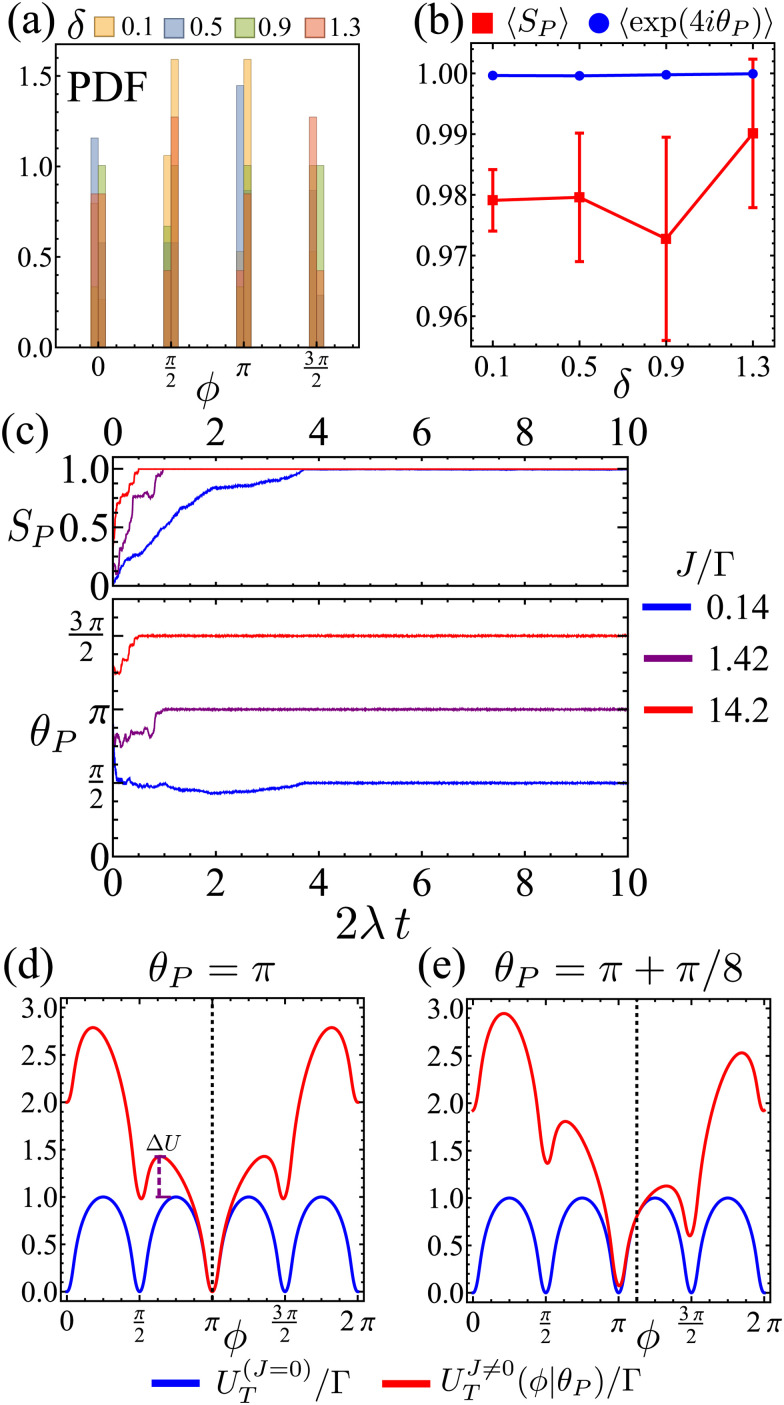
(a) Flocks' PDF of the global polar orientation *θ*_P_ for different Δ. (b) Mean flock scalar order parameter (red curve) and mean squarishness of *θ*_P_ (blue curve) as a function of *δ*. Notice how the scalar order parameter is significantly higher than the threshold of *S*_P_ = 0.85 used to determine its existence. (c) Time series of *S*_P_ (top) and *θ*_P_ (bottom) for different *J*/*Γ* and *δ* = 1.1. (d) and (e): mean field approximation of net angular potential (*U*_T_ = *ΓV* + *JV*_A_, red curves) and bare orientation potential (*ΓV*, blue) for an alignment potential towards the collective orientation *θ*_P_ (indicated by the dashed line). (d) *θ*_P_ = π. The effective energy barrier has increased by Δ*U*. (e) *θ*_P_ = π + π/8.

Therefore, at larger *J*, a flocking state arises (Fig. 12(b) and Movie 2, ESI†). Once the system enters a collective flocking state, the system acquires a global orientation that aligns with one of the four cardinal tumble-turn directions, independent of the value of *δ*. This can be clearly seen for *J*/*Γ* = 0.14 from the distributions of global orientations *θ*_P_ in Fig. 13(a) and from the squarishness of global orientations 〈exp(4*iθ*_P_)〉 depicted by the blue curve in Fig. 13(b). This implies that the microscopic anisotropic dynamics, aided by the alignment interaction, translates into a dynamical global one. This is not as obvious a result as it might appear at first. Although all particles tend to travel, on average, along one of the four preferred axes, they can, in principle, travel instantaneously along any direction, as seen in the angular distributions of solitary run-and-tumble turners (Fig. 5(b)). The alignment potential could have reinforced such fluctuations, resulting in broad distributions of global orientation. The fact that it does not highlights the strong interplay between local anisotropy and global order. Furthermore, this global order is characterized not only by its strong breaking of rotational symmetry, but also by a value of *S*_P_ → 1 (red curve in Fig. 13(b)). The time series of the order parameters show that the flocking state of run-and-tumble-turn particles has a persistent global orientation *θ*_P_ (Fig. 13(c)). This is in contrast to the usual flocking states, whose direction of motion fluctuates with time.^63^ For run-and-tumble-turn particles with strong *J*, the time needed to enter the flocking state is shorter (Fig. 13(c)). The flock locks in one of the preferred directions even at very high values of *J*/*Γ*, suggesting that the tumble-turn potential dictates the dynamics even when it ostensibly acts as a small perturbation.

The reason behind the above global order properties is that as particles of similar orientation meet, the alignment interaction reinforces their directions, further deepening the effective total angular potential *U*_T_ = *ΓV* + *JV*_A_ affecting the particles compared to the bare orientation potential *ΓV* (Fig. 13(d)). While the total orienting potential deepens, the angular noise remains fixed as suggested by eqn (8). This effect is robust against fluctuations. Indeed, if there is a fluctuation that causes neighbors to travel along *θ*_P_ (e*.g.*Fig. 13(e) for Δ*θ*_P_ = π/8) then the global minima does not shift (as long as |Δ*θ*_P_| < *π*/4) but the potential barrier is still larger, permitting the particle to resist their neighbors' fluctuation.

As particles cluster and a global order is formed, the turning times increase so much that the probability of having coordinated angular fluctuations capable of spanning the entire system is highly unlikely. As such, the angular sector of the energy landscape of the systems gets frozen in time, resulting in particles traveling perpetually along one of the four axes. In the absence of the orientation potential (namely, models of typical ABPs), this angular freezing of the energy landscape is much harder to achieve. Without the indentations caused by the orientation potential, particles could exhibit much higher angular fluctuations.

## 6 Conclusions

Inspired by self-motile cells in anisotropic environments and developments of colloidal inclusions in active nematics, we have studied the dynamics of a particle following a “run-and-tumble-turn” process, in which the particle is aware of its net alignment concerning the inherent frame of the environment and prefers to self-propel parallel to the four Cartesian axes. Through agent-based simulations of solitary run-and-tumble-turn particles following a Langevin equation including an orientation potential, the effects of noise and the width of the potential are explored at short and long time scales. Assuming that the strength of the potential is large compared to the strength of the orientational noise, one finds that the short-time effect is to renormalize the self-propulsion speed but keep the direction along the current axis. This process cannot induce a reversal in the direction of motion and thus only modifies the propulsive regime of the dynamics. At longer time scales, the noise can allow the solitary particle's orientation to overcome the angular potential barrier, inducing a right-angle turn. This process governs the long-time dynamics of the system and determines the transition time from propulsive to diffusive behavior.

We further explored how the angular potential's width affects the tumble-turn rate, the angular distribution's spread, and, therefore, how well collimated a beam of trajectories is. In particular, we learned that the width of the potential only alters the trajectories' squarishness at short-time (-length) scales. When looking at long-time (-length) scales, all trajectories are indistinguishable for the range of parameters we considered. With this in mind, for the sake of simplicity, we considered the limit of a very narrow potential that keeps particles traveling mostly parallel to the axes. In this limit, we developed a continuum model based on a four-component spinor that details the probability of having a particle traveling along one of the Cartesian axes. Solving this model numerically and comparing it with solitary agent-based simulations demonstrates a good qualitative match at long-time scales, even for wider potentials, as the change to diffusive behavior seems to be independent of the details that drive it. Furthermore, the spectrum and eigenvectors of this continuum model characterize the role of long- and short-length scales over time and how the dynamics shifts from propulsive to diffusive, and also how the latter behavior drives the system to a global isotropic behavior, despite its local anisotropicity. To study this transition further, we derived the model's hydrodynamic limit that only exhibits long wavelength features and solved it explicitly. Although exhibiting a purely diffusive regime, the model can drive and sustain global anisotropic behavior, albeit for intermediate times. The long-time anisotropicity decays inversely with time.

By adding short-range repulsion and alignment interactions to the Langevin equations, we showed that the anisotropic features of individual particles can be inherited by the global order of an ensemble of many interacting run-and-tumble-turn particles. Explicitly, we observed that, at sufficiently high alignment interactions, the system enters a flocking transition along one of the four preferred axes. The flocking state is found to have high orientational order, as well as a stationary orientation in contrast to the flocking of ABPs, which typically fluctuates. We justify that these properties emerge due to the alignment interaction reinforcing the orientation potential, leading to an exponential suppression of turning rates, the freezing of the angular sector of the energy landscape, and therefore, a highly protected macroscopic order.

We hope our results will expand the current literature on active random processes, particularly in processes explicitly presenting four-fold symmetric tumbles^50,53,54^ or in which keeping anisotropic features on the global scale is important, such as biological processes occurring in anisotropic environments or that exhibit inherent anisotropic features, as recent experiments in morphology have shown how anisotropic division in shrimp embryos leads to a macroscopic four-fold symmetry.^64^ While we are not aware of experiments that have directly studied the translation of anisotropic features on macroscopic length scales or global order, there are currently realizable experiments that could test these predictions. For example, movements of cells in patterned substrates^50,65^ could, in principle, replicate both the right-angled turns and the repulsive interaction. Similarly, there is evidence for bacteria swimming in a nematic liquid crystal exhibiting right-angled tumbles.^66^ Likewise, bristle-bots^67^ could be modified to exhibit right-angled turns. Since they are elongated, they would naturally exhibit aligning interactions at sufficiently high packing fractions.

Future work will be needed to fully characterize the flocking and MIPS transitions in the interacting case. Considering alignment interactions much stronger than the orientation potential may allow the run-and-tumble-turn to collectively abandon their square-like trajectories. Furthermore, adding frustration to the system is possible by incorporating more species with different preferred directions of motion, which may lead to novel collective dynamics. Future work should also study the interplay between run-and-tumble-turn particles and boundaries. These results can contribute to a better understanding of the mechanisms that help translate local anisotropies into macroscopic lengthscales and global orders, and apply them to similar morphological processes. Finally, provided the recent interest in motility strategies in bacteria, we hope that by highlighting this route from local active anisotropies translate to larger scales, we can contribute towards the development of control protocols of living systems and the engineering of their transport processes.

## Conflicts of interest

There are no conflicts to declare.

## Supplementary Material

SM-020-D3SM00589E-s001

SM-020-D3SM00589E-s002

## Figures and Tables

**Fig. 14:**
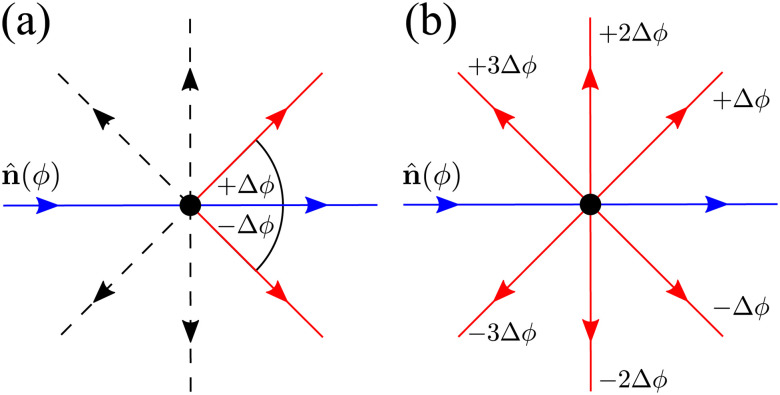
Diagrams depicting the two possible extension for *N* = 8. Blue arrows point towards the current direction of motion (specified by an orientation *ϕ*). Red arrows indicate a direction in which a turn is allowed. Dashed black arrows indicate directions in which the particle can propagate but, for this particular turning event, are not allowed. (a) Only neighbors extension: only turns in directions separated by ±Δ*ϕ* respect to *ϕ* are allowed. (b) All directions: all possible turns are allowed, except for turning back.

## Appendices

### Appendix A Numerical details

Particle simulations were written in C++ and optimized through OpenMP. Special functions (dilogarithm) were implemented through the GSL library. All runs are carried out with *N* = 1000 independent particles and use the following parameters (in simulation units): *v*_0_ = 1, *Γ* = 7.0, *D*_r_ = 1.0, a timestep of δ*t* = 10^−5^ and *δ*∈[0.1,1.3].

For the case of interacting particles, we also model *N* = 1000 particles of radius *r*_c_ = 1 in a box with periodic boundary conditions and size *L* = 79, resulting in a packing fraction of 0.5 approximately. Furthermore, to make the persistence length smaller than the size of the system, we use a self-propulsion speed *v*_0_ = 0.125. We keep *D*_r_ = 1 and *Γ* = 7.0. With this choice, *v*_0_/(*λL*) ∈ (0.06,0.16). The parameter values of the interacting potentials are *ε* = 10, *r*_A_ = 2.5, and *J* ∈ (0.1,100), though for most runs, we pick *J* = 1. Particles are initialized with random positions and orientations. The code was further optimized by establishing neighbor lists, which were constructed using a buffer distance of 3 particle radiuses over the alignment radius *r*_A_ and updated every *r*_c_ / (*v*_0_d*t*) time steps. This corresponds to the time (in time step units) needed for a particle to move 1 radius traveling in a straight direction.

Eqn (21) is numerically integrated in C using a third-order upwind finite difference scheme. This was done in a square lattice 1001 × 1001 with spacing δ*x* = 1. The parameters used are: *λ* = 0.01, *v*_0_ = 1, and timestep δ*t* = 0.001. The system is initialized in a Dirac delta-like initial condition with all probabilities having the same distributions.

### Appendix B Generalization to *N*-fold symmetry and the continuum limit

In this section, we generalize our theory to the case in which particles can turn in *N* different equally distributed directions, that is, equally separated by an angular difference Δ*ϕ* = 2π/*N*. We pay close attention to how isotropicity is recovered in the continuum limit, Δ*ϕ* → 0.

Depending on how many turns are accessible to a particle at an instant in time, there are at least two different approaches to this generalization: (i) allow the particles to turn only to neighboring directions, *i.e.*, only Δ*ϕ* turns, or (ii) allow the particles to turn to any other direction, with the exception of a direct reversal. For a graphical depiction, see Fig. 14. Each of these choices leads to a different continuum theory. In the following, we detail both extensions. In both derivations, particles can now travel along the *N* directions, characterized by their polar angles , and so the spinor of probabilities ***P*** of Section 3 must now have *N* components.

#### Neighboring directions only

We focus on an arbitrary component *P*_*ϕ*_(***r***,*t*), that is, the probability that the particle will travel along *ϕ* at position *r* at time *t*. In this extension, the particle continues to be subjected to the steering potential, which has had its argument rescaled in order for its period to match the new required symmetry *V*_N_(*ϕ*) = *V*(π*ϕ*/(2Δ*ϕ*)). This implies that turning can only be done by jumping to neighboring minima of the potential *ϕ* ± Δ*ϕ* (Fig. 14(a)) and the turning rate associated with these turns *λ*_N_ is rescaled. Indeed by eqn (7), , which leads to the new rate

A1

With this, we can now follow the same procedure as in Section 3 and write

A2

in which ***n̂***(*ϕ*) = cos(*ϕ*)***ê***_*x*_ + sin(*ϕ*)***ê***_*y*_ is the unit vector specifying the travel direction associated to *ϕ*. This identity simply states that *P*_*ϕ*_(***r***,*t*) equals the probability that at *t* − d*t* the particle travels along *ϕ* ± Δ*ϕ* and turns plus the probability of continuing to travel along *ϕ* and not turning. Expanding eqn (A2) for small d*t*, keeping terms up to first order, using eqn (A1), and defining *D*_r_ ≡ π^2^*λ*/4 leads to the dynamical equation for the arbitrary component *P*_*ϕ*_

A3

in which ***v***(*ϕ*) ≡ *v*_0_***n̂***(*ϕ*). Taking the continuum limit Δ*ϕ* → 0 leads to

A4 (∂_*t*_ + ***v***(*ϕ*)·**∇**)*P*_*ϕ*_ = *D*_r_ ∂_*ϕ*_^2^*P*_*ϕ*_,

which is simply the well-known Fokker–Plank equation for an active Brownian particle (ABP) with rotational diffusion *D*_r_. It is explicitly invariant under rotations, thus confirming the intuition that as Δ*ϕ* becomes smaller, the theory becomes isotropic.

#### Turns in all (but backward) directions

This extension drops the steering potential and allows the particle to turn in all possible discrete directions (except going backward, Fig. 14(b)) with a net turning rate *λ̃*. As this turning rate takes into account all possible turnings, it must be the sum of the turning rates for individual directions. For simplicity, we assume that *N* is even and that all turning directions have the same turning rate *λ*_N_. The case for an odd *N* is similar, though without the need to worry about direct reversals. With these assumptions and recognizing that a particle has *N* − 2 possible turning directions, we must have *λ*_N_ = *λ̃*/(*N* − 2). Taking into account these assumptions, eqn (A2) is modified to

A5

Expanding and keeping terms up to first order in d*t* leads to

A6

To take the continuum limit, let *N* → ∞ (equivalently Δ*ϕ* → 0), which makes the right hand side of eqn (A6) a Riemann sum

A7

with a similar expression for the *ϕ* − *j*Δ*ϕ* terms. This leads to the following continuum limit

A8

which is the kinetic equation for a traditional isotropic run-and-tumble process. As this equation is also explicitly rotationally invariant, this extension also leads to an isotropic theory as the number of allowed directions increases. Nevertheless, notice that if one assigns different turning rates to each direction, as in Appendix C, the integral in eqn (A8) would exhibit a non-uniform kernel, which would weight directions as measured in the laboratory frame and make this process different to the one found in ref. 35 and 36.

### Appendix C Unequal turning rate

The anisotropicity of the long wave theory, eqn (27) is subtle because it originated on the 4-fold symmetry of the underlying master equation, eqn (21). Since these dynamics do not break the symmetry between the four preferred directions (left, right, upwards, and downwards), the resulting hydrodynamic theory does not distinguish between the Cartesian axes, leading to isotropic diffusion in the lowest gradient (as it can be seen in eqn (27)). Indeed, the anisotropic features arise at a higher gradient order. While in the main text, we focused on the consequences of this effect, in this appendix, we show how explicitly breaking the symmetry between the preferred directions causes the system to be explicitly anisotropic in that it develops a proper diffusion tensor.

So far, we have considered that there is an equal probability rate of turning right or left than to turning upwards or downwards. This balance, however, is not a necessity of the dynamics. For example, if the four minima of the angular potential of our colloids have different heights, then by Krammer's formula, the particles would experience different tumble-turn rates. Such an effect, for example, can occur in the Janus colloid of ref. 53: the active force propelling the colloid modifies the potential that bounds it to its companion defect, which is responsible for its steering dynamics. As such, the minima of the potential at the planar or homeotropic sides have different heights. Although this effect is negligible at small activities, it can be important as activity increases. Inspired by the above, we consider a particle whose lateral turning rate, *λ*_L_, differs from its vertical one, *λ*_V_. Taking 1/(*λ*_L_ + *λ*_V_) as the unit of time and *v*_0_/(*λ*_L_ + *λ*_L_) as the unit of length, this results in eqn (24)–(26) being modified to

A9 ∂_*t*_*ρ* + **∇** · ***j*** = 0,

A10

A11 ∂_*t*_*χ* + **∇**_p_ · ***j*** = −2*χ*,

in which we have defined

A12

and α ≡ *λ*_V_/*λ*_L_. Proceeding as before, we obtain the following hydrodynamic theory

A13

Setting *α* = 1 results in the same expression as eqn (27). By rescaling lengths again by  we finally get

A14

This equation explicitly breaks the symmetry between axes. Moreover, the anisotropy now enters at the leading order in gradients. Indeed, we can readily read the diffusion tensor

A15

Beyond this anisotropic diffusion, eqn (A14) still presents higher order anisotropic terms originating in the rectangular-ness of the underlying dynamics.
